# Fidelity Assessment Checklist Development for Community Nursing Research in Early Childhood

**DOI:** 10.3389/fpubh.2021.582950

**Published:** 2021-05-14

**Authors:** Lubna Anis, Karen M. Benzies, Carol Ewashen, Martha J. Hart, Nicole Letourneau

**Affiliations:** ^1^Department of Pediatrics, Faculty of Nursing, Owerko Centre, Alberta Children's Hospital Research Institute, University of Calgary, Calgary, AB, Canada; ^2^Department of Pediatrics, Faculty of Nursing, University of Calgary, Calgary, AB, Canada; ^3^Faculty of Nursing, University of Calgary, Calgary, AB, Canada; ^4^Department of Community Health Sciences, Faculty of Nursing, Owerko Centre, Alberta Children's Hospital Research Institute, University of Calgary, Calgary, AB, Canada

**Keywords:** parenting, intervention, program, fidelity tools, checklist, measure, early childhood, ATTACH

## Abstract

Nurses play an important role in promoting positive childhood development via early interventions intended to support parenting. Despite recognizing the need to deliver vital parenting programs, monitoring fidelity has largely been ignored. Fidelity refers to the degree to which healthcare programs follow a well-defined set of criteria specifically designed for a particular program model. With increasing demands for early intervention programs to be delivered by non-specialists, rigorous yet pragmatic strategies for maintaining fidelity are needed. This paper describes the step-by-step development and evaluation of a program fidelity measure, using the Attachment and Child Health (ATTACH™) parenting program as an exemplar. The overall quality index for program delivery varied between “very good” to “excellent,” with a mean of 4.3/5. Development of checklists like the ATTACH™ fidelity assessment checklist enables the systematic evaluation of program delivery and identification of therapeutic components that enable targeted efforts at improvement. In future, research should examine links between program fidelity and targeted outcomes to ascertain if increased fidelity scores yield more favorable effects of parenting programs.

## Introduction

Parents influence children's affective and cognitive development, with lifelong impacts (1, 2). Nurses play an important role in intervening early to support parenting and promote healthy child development (3, 4). Despite recognizing the need to deliver vital parenting programs, monitoring fidelity has been historically ignored (5, 6). Fidelity refers to the extent to which a healthcare program follows an explicit set of criteria specifically designed for its particular program model (7–9). However, defining and operationalizing program fidelity for parenting programs is difficult due to their interactive and dynamic nature (10, 11). Attempts by program developers to assure adherence to their programs include the creation of training and protocol manuals, but these alone may not be sufficient to ensure fidelity of implementation (8, 12). With increased demands for early intervention to be widely delivered by non-specialists, rigorous yet pragmatic strategies for maintaining program fidelity are needed.

In the United States and Canada, the development and implementation of parenting programs to bolster healthy development of children has increased during the last 20 years (13). There is a growing awareness of the significance of developing and applying fidelity measures to evaluate the implementation of such programs (12, 14, 15). However, practical guidelines and exemplars are lacking. This paper describes how to develop and conduct an evaluation of a program fidelity checklist, including the step-by-step developmental process our research team used for the Attachment and Child Health (ATTACH™) parenting program. ATTACH™ is a psycho-educational parenting program that fosters parental reflective function (RF), the ability of parents to envision mental states in themselves and their children to promote healthy child development (16, 17). Compared to routine care, ATTACH™ has been shown to be effective in randomized controlled trials and quasi-experimental studies (16, 17); however, program fidelity assessment was needed to contextualize results and assure ongoing internal validity in support of wider implementation. Thus, the purpose of our paper is to demonstrate the step-by-step process for development of a fidelity checklist, specifically for ATTACH™, and to provide preliminary data on its utility.

### Background

The practice of assessing fidelity in community-based interventions was adopted from trials of pharmacotherapy (drug) trials, in which strict adherence to protocol is a critical requirement (18, 19). In contrast to drug trials, systematic evaluation of program fidelity of psycho-educational programs, such as parenting programs, is more difficult due to the dynamic and often highly individualized interactions between facilitator and parent (20, 21). It is also more challenging to ascertain the therapeutic elements of parenting programs (22) as they tend to be tailored to parents' individual needs and preferences. However, tailoring a program does not mean that the facilitator may extemporize during the program administration; rather, program elements that are standardized vs. customized must be clearly defined and monitored (22, 23). Stated simply, facilitators need to be assessed on whether they delivered the program by using judgment and discretion appropriately.

Evaluation of program fidelity answers the following question: Did facilitators deliver the program as intended? (24). Strategies such as reviewing audio- or video-taped intervention sessions or direct observations to detect any diversions in program delivery have been recommended (25, 26). While helpful, these strategies offer insufficient guidance for rigorous evaluation of fidelity. The National Institutes of Health (NIH) Behaviour Change Consortium identified five elements to promote program fidelity (i.e., design, training, delivery, receipt, and enactment) (18, 27, 28); however, simply including the five steps does not ensure fidelity (14, 28, 29). The five steps offer little specific direction to the: (1) processes of conducting fidelity assessment, (2) determining which types of assessments are appropriate for a given program, or (3) how to define degree of adherence to and any deviations from program protocols (24, 30).

Assessing program fidelity includes consideration of content and process (20, 30–32). Content fidelity (or adherence) refers to the degree to which each main element is implemented as intended and if there are unplanned elements delivered (30, 31). Process fidelity (or competence) refers to the degree to which effective communication skills are used in response to facilitator and participant needs and situations, and essentially how well each intervention element is delivered (20, 21, 24, 30, 32–34). While adherence refers to the quantity of recommended behaviors, process refers to skillfulness in implementation of intervention (12, 35).

The cost and time required to develop a fidelity measure, training raters to code the intervention sessions, and establishing inter-rater reliability between the coders, contribute to the lack of reports of the systematic assessment of program fidelity. Although nurses may benefit from using extant program evaluation measures, instruments created for one program may not be promptly adaptable to others (36) as evaluation of program fidelity must be tailored to the program being tested (12). Additionally, nursing interventions are increasingly being delivered by other health professionals (37, 38); therefore, it is crucial to describe a step-by-step developmental process for a fidelity checklist that can be effectively used by many professionals.

How fidelity is measured and how checklists are developed matters a great deal in terms of assessing adherence to any kind of practice (39–41). Psycho-educational parenting programs like ATTACH™ require a great deal of mutual interaction (between the facilitator and the participant), which contributes to the skillful delivery of program elements; it may also pose difficulty in training facilitators and examining the quality of program delivery (42, 43). A checklist to assess program fidelity may facilitate a systematic evaluation of program delivery as well as facilitators' training and help with interpretation of intervention effects.

## Materials and Methods

To develop and test the fidelity checklist for ATTACH™, we evaluated existing measures for monitoring fidelity of parenting programs. We determined their applicability to the ATTACH™ program and identified challenges that needed to be resolved for our fidelity checklist. Then we created the ATTACH™ fidelity checklist by tailoring standard recommendations (18, 24, 30, 32, 34, 44–47) to ATTACH™'s guiding theory and program structure.

### The Attach™ Program

Details of the ATTACH™ program and the guiding theory are published elsewhere (16, 17). Briefly, ATTACH™ is a 1-h, 10–12 session, face-to-face intervention with dyadic (mother and infant) and triadic (mother, infant, and co-parent) elements. ATTACH™ is designed to help parents bolster a skill called parental Reflective Function (RF), the capacity of parents to think about mental states (thoughts, feelings, and intentions) in themselves and their children, and to consider how their mental states might affect their children to regulate behavior (16, 17). Parental RF is distinguishable from many related terms including mindful parenting (48–50), mindblindedness (51), empathy (52), insightfulness (53), and mind mindedness (54). ATTACH™ can be delivered by nurses or other health professionals with an undergraduate degree in health sciences, social work, psychology, sociology, or some post-secondary education that relates to child welfare. During weekly ATTACH™ sessions, that involve review of parent-child play sessions, and discussions of real-life and hypothetical or made-up stressful social situations, the facilitator helps the parents learn new RF skills, accomplished through leading by example, asking questions and practicing RF skills. The ATTACH™ studies were approved by an appropriate institutional review board.

### Review of Extant Parenting Program Fidelity Measures

To examine utility for the evaluation of ATTACH™, twelve fidelity measures were reviewed to determine if and how content, process, adherence and competence fidelity were assessed (Table 1). All measures included “adherence” to program content elements as a part of the measures. Additionally, fidelity checklist developers of the Leadership Observation Tool (45), Common Sense Parenting Trial (60), Getting Ready Project (61), Family Check-Up (44, 47) and EARLY ALLIANCE prevention trial (24) included competence to process elements or as a part of their checklists to capture process skills of facilitators. Furthermore, “participants' responsiveness” was also taken into consideration in the Chicago Parent Program fidelity tool (14), and the Fidelity of Implementation Rating System (FIRS) (57, 58, 65) to ensure the program was received and understood by the participants. Finally, an overall score was assigned to rate the “overall quality index of program delivery” by using a specific criterion in the Chicago Parenting Program checklist (14).

**Table 1:** Review of extant fidelity tools for parenting intervention.

	**Fidelity tools**	**Description of tool/intervention**	**Fidelity elements**
1.	The Parent Programme Implementation Checklist (55)	A simple, brief and generic observational tool for assessment of implementation fidelity of group-based parent programmes.	Checklist focusing on Adherence, Exposure or length of session, Quality of program delivery, Participant responsiveness, and Program differentiation.
2.	Implementation fidelity tool for a school-based parenting program for low-income families (14)	Chicago Parent Program (CPP): a 12-session, video and group-based parenting skills training program that improves parenting skills and confidence and reduce behavior problems in young children 2–5 years old.	Content fidelity for intervention design, training interventionist; Delivery; Receipt; Enactment.
3.	Quality assurance/fidelity checks for Triple P - Positive Parenting Program (56)	Triple P - Positive Parenting Program: a parenting intervention to enhance the knowledge, skills, and confidence of parents, and reduce the behavioral and emotional problems in children and adolescents.	Session Checklists; Accreditation of practitioners.
4.	Fidelity of Implementation Rating System (FIR) (57–59)	Evaluation of 5 dimensions of competent adherence to Oregon model of Parent Management Training (i.e., knowledge, structure, teaching skill, clinical skill, and overall effectiveness).	Adherence to the intervention's core content components; Competent execution using accomplished clinical and teaching practices.
5.	Implementation assessment of Common Sense Parenting trial (60)	Common Sense Parenting: a parenting program to improve the transition to high school.	Intervention training protocol; Session checklist focusing on Adherence; Exposure or length of session; Quality of program delivery; Participant satisfaction.
6.	Treatment Fidelity in Evidence-Based Parent Training Programs for Externalizing Disorders in Children and Adolescents (29)	Evidence-Based Parent Training Programs for Externalizing Disorders in Children and Adolescents.	Aspects of intervention design, intervention delivery, training providers, and assessment of participant receipt of intervention and enactment of treatment skills.
7.	Fidelity measurement of a relationship-based school readiness intervention: (61)	Getting Ready project: an integrated, multi-systemic intervention that promotes school readiness through parent engagement for children from birth to age five.	Adherence, quality of intervention delivery, differentiation between groups, and participant responsiveness.
8.	Leader Observation Tool (LOT): A process skills treatment fidelity measure for the Incredible Years parenting programme (45)	Incredible Years Parenting Program: a group-based parenting program to improve parenting behavior and reduce child behavior problems.	Process fidelity including listening, empathy, physical encouragement, positive behavior.
9.	Intervention fidelity tool for the EARLY ALLIANCE prevention trial (24)	EARLY ALLIANCE, a prevention trial currently testing the effectiveness of family, peer, and school interventions to promote competence and reduce risk for conduct disorder, substance abuse, and school failure.	Content fidelity and process fidelity (including listening, respect, gestures, tone, instructions, questioning, preparedness on a 4-point Likert scale).
10.	Family Check-Up: (44, 47)	Family Check-Up (FCU): a brief, personalized parenting intervention designed to improve youth adjustment by motivating use of effective parenting skills.	Adherence to the FCU model and the quality of the delivery (assessed on a 9-point scale (needs work: 1–3, competent work: 4–6, excellent work: 7–9).
11.	Attachment and Biobehavioral Catch-up intervention (62)	Attachment and Biobehavioral Catch-up (ABC): a 10-session, home visiting parenting intervention developed to address the regulatory and attachment problems of children experiencing early adversity, including neglect.	Process fidelity measure.
12.	Parent-Child Interaction Therapy (63, 64)	Parent-Child Interaction Therapy (PCIT): a short-term treatment that enhances the parent–child relationship and utilizes *in vivo* coaching to promote parent skills.	Process fidelity measure.

One of the major challenges or limitations with the extant fidelity measures was the vague boundary between fidelity to content elements (or adherence) and fidelity to process elements (or competence), expected to be consistent with the theoretical model of the program (24, 45). Most of the program fidelity measures we reviewed were focused on monitoring a skill set or approach, a process element to measure content fidelity (24, 56, 62–64). Because the contents and the conceptual model of the ATTACH™ were closely related to the theoretical underpinnings of parental RF, in addition to the adherence, we needed to directly evaluate the use of the principles of parental RF deemed essential to the program. For example, exploration of “parental RF” representations was expected to be consistent with the theoretical model of ATTACH™ in each element of intervention.

Another challenge was the inattention to identifying unplanned program elements, an element of process fidelity (56). To describe the relationships between intervention delivery and outcomes accurately, it was essential to assess not only what and how many program elements were implemented but also whether and how many unplanned elements were delivered (57, 58, 65). This knowledge would improve training, monitoring and specific retraining for facilitators' adherence to the program protocol.

Adequate pacing, which allows the facilitators to deliver each step of intervention in a certain duration of time without rushing or dragging, is another important aspect of process fidelity (66). However, evaluation of facilitators' pacing has never been included in the fidelity measures of parenting measures that we reviewed. Although one fidelity tool (55) required calculating facilitator's and participant's talk time, talk time did not include facilitators' pacing through the program steps, which was necessary for our purposes. Also, cut-offs/criterion ratings for satisfactory levels of adherence are not frequently used in the parenting intervention fidelity literature. Although Caron et al. (67) described certification levels for the ABC fidelity measure, the decisions on the cut-offs appeared to be arbitrary. To over come these challenges, we focused on broader fidelity measure literature from psycho-educational interventions of non-parenting programs for guidance e.g., Song et al. (66), Miller et al. (68), Miller et al. (69) and Miller et al. (70).

### ATTACH™ Fidelity Assessment Checklist

In developing the ATTACH™ Fidelity Assessment Checklist, we extracted elements from the extant measures that best met fidelity requirements for our ATTACH™ fidelity checklist. We created a dictionary of operational definitions of the program elements or checklist items that is available on request. This includes, but is not limited to the definitions of: thoughts and feelings: liked, disliked, and interesting moments; the hypothetical and real life situations; and rushing, connecting, disconnecting, dragging, resistant, going along, and pacing. For example, during the video feedback component of the intervention, the facilitator invites the parent to choose a part of the free play interaction they like or found interesting to ascertain what the parent is *thinking* (e.g., mother thinks her child is smart as child shakes the rattle) and *feeling* (e.g., mother describes pride) and what the parent thinks their baby is thinking (e.g., child wants to shake the rattle) and feeling (e.g., child is happy) in those moments.

#### Adherence to Program Content Elements

To explicitly define and evaluate delivery of theoretical components of the ATTACH™, we determined the program elements based on the guiding principle and prescribed steps of the program. The five steps included in the dyadic sessions and the four steps included in the triadic sessions had a total of 30 and 26 elements, respectively, as shown in Table 2. Each content element included several prescribed questions as shown in Tables 3, 4. To evaluate adherence to program content elements, each program component was coded as *Yes* (attempted = implemented as intended) or *No* (not attempted = never asked or failed to perform). Additionally, to evaluate the overall adherence to program content elements, the occurrences of *Yes or No* elements were simply summed. These numbers were then divided by the total number of elements (31) and multiplied by 100 to compute percentages; a higher score in the *Yes* category reflected higher program content fidelity. An intervention is typically regarded as implemented with high fidelity when there is >80–90% adherence to content (45, 62, 66, 71). Therefore, to be considered satisfactory, content fidelity was expected to be 90% or higher for *Yes* categor*y*, or 10–20% or lower for *No* category. Each element was expected to be treated as equal in importance at this stage of creation of the checklist.

**Table 2:** ATTACH™ program content outline for dyadic and triadic sessions.

**ATTACH sessions**	**Content by session**
Dyadic sessions	Step #1: Introduction to RF and What to Expect During the ATTACH Intervention (2 elements) Step #2: 3–5 min Free Play and Video Feedback (Parent: 1 like, 1 dislike – Facilitator: 1 interesting = intrusive) (16 elements) Step #3: Hypothetical Situation: Introduced by Facilitator (4 elements) Step #4: Real Life Situation: Introduced by Parent (4 elements) Step #5: Debriefing (4 elements) Total = 30 elements^**^
Triadic sessions	Step #1: Introduction to RF for the Co-parent (2 elements) Step #2: 5–6 min Free Play and Video Feedback (Parent: 1 like, 1 dislike – Co-Parent: 1 like, 1 dislike - Facilitator: 2 interesting = 1 parent, 1 co-parent – intrusive for both) (16 elements) Step #3: Hypothetical Situation: Introduced by Facilitator (4 elements) Step #4: Debriefing (4 elements) Total = 26 elements^**^

** *Content elements are described in detail in Tables 3, 4*.

**Table 3:** ATTACH™ fidelity checklist for dyadic sessions.

Location: ______________________________________________________________	
Parent's name: _____________________ Child's name: ________________________	
Child's age: ____________________________________________________________	
Session date and time: _____________________ Facilitator: _____________________	
**A. Adherence to intervention content elements**	
**1. Introductions**:	
(a) Facilitator describes the purpose of the intervention session and what kinds of questions will be asked and discussed (when applicable): □ Yes □ No (b) Facilitator asks about the past week: □ Yes □ No **2. 3-min free play and video feedback:** 3- min video completed: □ Yes □ No *Video Feedback:* *Like (Session 1,2,3,4)/ Connecting (Session 5,6,8 and 10):* □ Yes □ No - Parent's thoughts: □ Yes □ No - Parent's feelings: □ Yes □ No - Child thoughts: □ Yes □ No - Child's feelings: □Yes □ No *Dislike (Session 1,2,3,4)/ Disconnecting (Session 5,6,8 and 10):* □ Yes □ No - Parent's thoughts: □ Yes □ No - Parent's feelings: □ Yes □ No - Child thoughts: □ Yes □ No - Child's feelings: □ Yes □ No *1 Interesting moment (Session 1,2,3,4)/ 2 Interesting moments Session 5,6,8 and 10*: *□ Yes □ No* - Parent's thoughts: □ Yes □ No - Parent's feelings: □ Yes □ No - Child thoughts: □ Yes □ No - Child's feelings: □Yes □ No **3. Reflective exercises:** Facilitator introduces the hypothetical situation to parent and ask her/ him to reflect on it. Facilitator asks everyone's thoughts & feelings x 2 to explore two distinct states using same hypothetical situation. *Hypothetical Situation:* □ Yes □ No First round (thoughts and feelings): □ Yes □ No Second round (thoughts and feelings): □ Yes □ No **4. Real life situation:** Parent introduces a real-life stressful parenting situation to facilitator and reflects on it. Facilitator asks everyone's thoughts & feelings x 2, first in actual life situation and then in conjectural real-life situation. *Real Life Situation*: □ Yes □ No First actual real-life Situation: □ Yes □ No Second conjectural real-life situation: □ Yes □ No **5. Debriefing and additional support:** Facilitator asks the parent how they thought the session went and discuss any issues that come up. (a) Session debrief: □ Yes □ No (b) Social support provided: □ Yes □ No □ N/A(Yes or N/A responses are counted as 1) (c) Next appointment booked? □ Yes □ No (d) Walked the parent out? □ Yes □ No	**Additional comments**: (e.g., nature of support received, unexpected events, notes about visit to follow up next time)
Total count of ‘Yes’ items: _________/30 items; _____________%	
Total count of “No' items: _________/30 items; _____________%	
*To be considered satisfactory, content fidelity is expected to be 80-90% or higher for Yes category, or 10-20% or lower for No category*.	
**B. Adherence to process elements or competence**	
Note: The facilitators are expected to take notes and repeat/ restate/ paraphrase/ and ask timely probing questions for the video feedback, the hypothetical situation, and the real-life situation components of the session = 24 items.	
1. *Repeat/restate/paraphrase/timely probing questions: _________/24 items; ___________%	
2. **Skipped opportunities:** _____________/24 items; ___________%	
*To be considered satisfactory, process fidelity is expected to be 70-90% or higher for repeating/restating/ paraphrasing/ using timely probing questions, and 10-30% or lower for skipped opportunities*.	
*Repeat/restate (simply repeat to what the participant has said using some or all the same words) *Paraphrase (change or add to what the participant has said in a significant way to speculate his or her meaning, something that he or she has not yet said directly) where appropriate *Timely probing questions (when the participant was having a hard time to reflect)	
**3. Pacing:** Skill to deliver each step of intervention in 45–60 min without rushing or dragging	□ 1 – Too short (<20 min) □ 2 – Adequate (45–60 min) □ 3 – Too long (>60 min)
**4. Participant's responsiveness** 1 (resistant) to 3 (going along)	□ 1 □ 2 □ 3
**Overall quality index of intervention delivery**	
1. Content Fidelity =	
2. Process Fidelity:	
a) Repeat/restate/ paraphrase/ timely probing questions (ratio) =	
b) Skipped (ratio) =	
3. Pacing =	
4. Participant's responsiveness =	
5. Overall Quality Index on a Likert scale of 1 (poor) to 5 (excellent) =	
1 = Poor	
2 = Average	
3 = Good	
4 = Very good	
5 = Excellent	

**Table 4:** ATTACH™ fidelity checklist for triadic sessions.

Location: _____________________________________________________________	
Parent's name: _____________________ Child's name: ________________________	
Child's age: ___________________________________________________________	
Co-parent's name: ______________________________________________________	
Session date and time: _____________________ Facilitator: ____________________	
**A. Adherence to intervention content elements**	
**1. Introductions:**	
(a) Facilitator describes the purpos of the intervention session and what kinds of questions will be asked and discussed to the co-parent: □ Yes □ No (b) Facilitator asks about the past week: □ Yes □ No **2. 5–6-min free play and video feedback:** 5–6-min video completed: □ Yes □ No *Video feedback for parent:* *Like: □ Yes □ No* - Parent's thoughts: □ Yes □ No - Parent's feelings: □ Yes □ No - Child's thoughts: □ Yes □ No - Child's feelings: □ Yes □ No *Dislike: □ Yes □ No* - Parent's thoughts: □ Yes □ No - Parent's feelings: □ Yes □ No - Child's thoughts: □ Yes □ No - Child's feelings: □ Yes □ No *Video feedback for co-parent:* *Like: □Yes □ No* - Co-parent's thoughts: □ Yes □ No - Co-parent's feelings: □ Yes □ No - Child's thoughts: □ Yes □ No - Child's feelings: □ Yes □ No *Dislike: □ Yes □ No* - Parent's thoughts: □ Yes □ No - Parent's feelings: □ Yes □ No - Child thoughts: □ Yes □ No - Child's feelings: □ Yes □ No *Video feedback for both the parent and co-parent:* *Interesting: □ Yes □ No* - Parent's thoughts: □ Yes □ No - Parent's feelings: □ Yes □ No - Co-parent's thoughts: □ Yes □ No - Co-parent's feelings: □ Yes □ No - Child's thoughts as described by the parent: □ Yes □ No - Child's feelings as described by the parent: □ Yes □ No - Child thoughts as described by the co-parent: □ Yes □ No - Child's feelings as described by the co-parent: □ Yes □ No **3. Reflective exercises:** Facilitator introduces the hypothetical situation to the parent and the co-parent and asks them to reflect on the situation. Facilitator asks everyone's thoughts & feelings x 3 to explore three distinct states using same hypothetical situation. *Hypothetical Situation:* □ Yes □ No Parent-led first round (thoughts and feelings): □ Yes □ No Co-parent led second round (thoughts and feelings): □ Yes □ No Parent and co-parent collaborated third round (thoughts and feelings): □ Yes □ No **4. No real – life situation** to avoid any potential conflict.	**Additional comments**: (e.g., nature of support received, unexpected events, notes about visit to follow up next time)
**5. Debriefing and additional support:** Facilitator asks the parent and the co-parent how they thought the session went and together they discuss any issues that came up. (a) Session debrief: □ Yes □ No (b) Social support provided: □ Yes □ No □ N/A (Yes or N/A responses are counted as 1) (c) Next appointment booked? □ Yes □ No (d) Walked the parent out? □ Yes □ No	
Total count of ‘Yes’ items: _________/26 items; _____________%	
Total count of “No' items: _________/26 items; _____________%	
*To be considered satisfactory, content fidelity is expected to be 80-90% or higher for Yes category, or 10-20% or lower for No category*.	
**B. Adherence to process elements or competence**	
Note: The facilitators are expected to take notes and repeat/ restate/ paraphrase/ and ask timely probing questions for the video feedback, and the hypothetical situation components of the session = 20 items.	
**Adherence to process elements**	
1. *Repeat/restate/paraphrase/timely probing questions: _________/20 items; ___________%	
2. **Skipped opportunities:** _____________/20 items; ___________%	
To be considered satisfactory, process fidelity is expected to be 70-90% or higher for repeating/restating/ paraphrasing/ and using timely probing questions, and 10-30% or lower for skipped opportunities. *Repeat/ restate (simply repeat to what the participant has said using some or all the same words) *Paraphrase (change or add to what the participant has said in a significant way to speculate his or her meaning, something that he or she has not yet said directly) where appropriate. *Timely probing questions (when the participant was having a hard time to reflect)	
**Pacing:** Skill to deliver each step of intervention in about an hour without rushing or dragging	□ 1 – Too short (<20 min) □ 2 – Adequate (45–60 min) □ 3 – Too long (>60 min)
**Participant's responsiveness** 1 (resistant) to 3 (going along)	□ 1 □ 2 □ 3
**Overall quality index of intervention delivery**	
1. Content Fidelity =	
2. Adherence to Process Elements:	
a) Repeat/restate/ paraphrase/ timely probing questions (ratio) =	
b) Skipped (ratio) =	
3. Pacing =	
4. Participant's responsiveness =	
5. Overall Quality Index on a Likert scale of 1 (poor) to 5 (excellent) =	
1 = Poor	
2 = Average	
3 = Good	
4 = Very good	
5 = Excellent	

#### Adherence to Process Elements or Competence

For the ATTACH™ fidelity checklist, we adapted the concepts of individual process skills largely from the Leadership Observation Tool (LOT) (45), the Common Sense Parenting Trial (60), the Getting Ready Project (61), and the EARLY ALLIANCE prevention trial (24). According to the ATTACH™ protocol, the facilitator was expected to maintain a positive communication behavior (for example, repeat or restate, paraphrase, and asking probing questions) by making notes for each component of the program, while using higher level of RF skills. For fidelity purposes, the occurrences of attempted communication behaviors were then counted followed by computing percentages. To be considered adequate, process fidelity or competence was expected to be 70–90% or higher, and 10–30% or lower for skipped opportunities, as suggested by literature (45, 62, 66). It deemed important to take both attempted communication behaviors and skipped opportunities into consideration when rating competence or adherence to process elements for accuracy purposes (45, 62, 66). A skipped opportunity is defined as the facilitator's missed opportunities to repeat/restate, paraphrase, or ask timely probing questions, e.g., asking about child's thoughts and feelings.

#### Pacing of the Program Delivery

For ATTACH™ to be paced adequately, the facilitator was expected to deliver each of the five steps in dyadic sessions, and four steps in triadic sessions in 45–60 min. We analyzed video-recorded sessions from pilot studies to determine recommended durations for the steps (17). For example, the duration of Step 2 may vary based on the time required to establish rapport with the participant; we expected this step to last at least 10 min to investigate all perspectives of the parent's representations of RF and should not last more than 20 min so that the remaining steps are completed without rushing. A 3-point rating scale from 1 (too short) to 3 (too long) was employed to rate the actual duration of the session as compared to the recommended duration range (55, 60, 66). Overall adherence to pacing was calculated by using a mean score of the ratings.

#### Participants' Responsiveness

Although the participants' responsiveness to the program is an aspect distinct from facilitators' adherence or competence, participants' acceptance of a program may impact fidelity (61). For example, if the participant is hesitant to further discuss reflective questions, this may hinder achieving the goals of the program. Others considered participants' responsiveness to be a potent mediator of the relationship between program or practice fidelity and participant outcomes (72, 73). For our checklist, we created items for this assessment as a determinant of fidelity, based on the concepts used in the Chicago Parent Program fidelity tool (14), and FIRS (57, 58, 65) to assess the degree to which ATTACH™ was received and understood by the participant. We used a Likert scale ranging from 1 (resistant) to 3 (going along) to rate participants' responsiveness (68).

#### Overall Quality Index of Program Delivery

The overall quality index included an overall assessment of the program delivery after getting a sense of the entire session by reviewing to the video-recorded session for adherence intervention content elements, competence, pacing and participants' responsiveness (14, 66, 68). This overall quality index was used to rate the quality of program delivery employing a predetermined criterion. The criterion was focused on facilitators' adherence to the program content elements and competence in delivering the program elements, without putting too much emphasis on frequencies of specific behaviors (14, 66, 70).

As described by (69, 70), typically this quality index evaluation included the extent to which the facilitator balanced their use of positive communication skills with their discretion in delivering the program on a 5-point Likert scale ranging from 1 (poor) to 5 (excellent). Each rating was defined e.g., overall quality index was expected to be rated 5 when the facilitator strictly adhered to the program components, by utilizing higher levels of clarification skills (e.g., repeating, reframing), making gentle and timely transitions, using probing questions ranging from fact to emotion probing, and missing few opportunities. Facilitators' overall quality index was expected to be rated 1 when they hardly used clarification skills (e.g., repeating, paraphrasing, reframing) probing questions, opportunities were often missed to explore further or promote RF, and the entire session felt like a question-answer session.

#### Decisions About Percentages and Ratings

The decisions about the percentages and ratings were adopted from fidelity assessments of psycho-educational interventions of both parenting and non-parenting programs (24, 30, 44, 45, 55, 66, 71). The percentages (used in the overall adherence to intervention content and process elements) and ratings (used in the pacing, dyad responsiveness, and over quality index of intervention delivery) were assessed in the ATTACH™ fidelity checklist. In deciding the number of points to be included in our rating scale (e.g., binary or Likert-type scale), we needed to carefully consider whether the rating scale would allow for raters to demonstrate their ability to differentiate among the set of behavior and activities to be coded (74). We also considered and whether such a detailed rating is useful and purposeful in evaluating fidelity. Taking these aspects into consideration, we elected to use 3-point ratings on the Likert scale items for pacing and participant's responsiveness, and 5-point ratings on the Likert scale items for overall quality index of program delivery (66).

### Application of the ATTACH™ Fidelity Assessment Checklist

The resultant fidelity checklist was implemented as part of testing of the ATTACH™ program (see Tables 3, 4). There were two facilitators, authors LA and MH, responsible for program delivery. The facilitators completed comprehensive competency-based training, relying on role playing and skill demonstration and employed training manuals. The facilitators delivered the ATTACH™ sessions, which were video-recorded, and completed ATTACH™ visit forms. The video sessions were assessed to examine fidelity of the ATTACH™ sessions.

### Data Analysis

To select a representative sample, we selected video recordings from one dyadic and one triadic session for each of 18 participants who completed ATTACH™, by selecting alternate sessions from both dyadic and triadic sessions. Thus, we examined 36 videotaped sessions (from pilot studies #1 and #2). Two trained raters independently coded session fidelity using the ATTACH™ fidelity checklist. We assessed inter-rater agreement between the coders by computing intraclass correlation coefficients [ICC; Type A (two-way mixed) using an absolute agreement definition; (55, 75)] using IBM SPSS Statistics version 26 software. Coders were also asked to comment on the utility of the checklist.

## Results

Table 5 shows an example of three ATTACH™ sessions assessed using the ATTACH™ fidelity assessment checklist. We selected the six sessions from three participants (both dyadic and triadic) to illustrate the full scope of assigned ratings and how the checklist may be used for monitoring fidelity and training. For example, for the first participant (No. 4), the overall quality index rating was 4.5 for both dyadic and triadic sessions. This rating was based on the 96–100% adherence on content and process fidelity, adequate pacing of program delivery, and high rating on participant's responsiveness for dyadic session. For the second participant (No. 11), an overall rating of 5 for dyadic session reflected completion of all elements of content fidelity (100%), process elements (100%) with no missed opportunities, completion of the session within time (adequate pacing), and high rating on participant's responsiveness for triadic session. For the third participant (No. 15), the overall rating was 4 for dyadic session, and adherence to content fidelity was 93% meaning that the facilitator skipped two items and took longer than the protocol required to complete the dyadic session.

**Table 5:** Evaluation of 6 sessions from 3 participants using the ATTACH™ fidelity checklist.

	**Intervention sessions**					
	**No. 4**		**No. 11**		**No. 15**	
	**Dyadic session**	**Triadic session**	**Dyadic session**	**Triadic session**	**Dyadic session**	**Triadic session**
**Adherence to intervention content clements**						
Elements implemented as intended (Yes)	96%	96%	100%	96%	93%	100%
Elements not implemented as intended (No)	4%	4%	–	4%	7%	–
**Adherence to process elements or competence**						
Repeat/paraphrase/probing questions	96%	95%	100%	100%	100%	100%
Skipped opportunities	4%	5%	–	–	–	–
Pacing (rating) 1 = too short, 2 = adequate, 3 = too long	Mean = 2	Mean = 2	Mean = 2	Mean = 2	Mean = 3	Mean = 2
Participant's responsiveness (rating) 1 (resistant) to 3 (going along)	Mean = 3	Mean = 3	Mean = 3	Mean = 3	Mean = 3	Mean = 3
Overall quality index of intervention delivery (rating) 1 (poor) to 5 (excellent)	Mean = 4.5	Mean = 4.5	Mean = 5	Mean = 4.5	Mean = 4	Mean = 5

Table 6 indicates the adherence and competence of the ATTACH™ facilitators. Under adherence to program content elements, the percentage for implemented as intended was 96% for both dyadic and triadic sessions. The percentages for elements implemented as not intended was low, as expected, between 2 and 3%. Under adherence to program process elements, the fidelity for repeat/paraphrase/probing was 99%, while skipped opportunities was 0 as expected. For pacing, the mean was 2.16 for dyadic and 2.3 for triadic sessions out of possible 3. For participant responsiveness the mean was 3 for both dyadic and triadic sessions out of possible 3. The overall quality index for program delivery varied between “very good” to “excellent,” with a mean of 4.3/5. The coders who coded the videos provided positive feedback on the checklist describing that they found user friendly. There were minor changes to address clarifications requested by the coders. For example, our preliminary checklist only included what the parent like/disliked about the play session. However, coders noticed that in later sessions the facilitator changed from asking what the parent like/disliked about the play session to moments the parent felt they were connected/disconnected with their child. We revised our checklist accordingly. Evaluation of the sessions from the ATTACH™ from earlier to later participants showed that the facilitators improved over time, as demonstrated by the improved fidelity ratings. The ATTACH™ fidelity assessment checklist could be used for training and assessing fidelity. The ICC for agreement between the two coders for the three elements of the fidelity checklist (adherence to the ATTACH™ content elements, process elements, and overall quality index) was good to excellent (ICC = 0.85–1.00), suggesting strong inter-rater agreement.

**Table 6:** Facilitators' compliance assessment by using ATTACH™ fidelity checklist.

		***n***	**%/Mean (SD)**
**Adherence to intervention content clements**			
Elements implemented as intended (Yes)	Dyadic sessions	18	96%
	Triadic sessions	18	96%
Elements not implemented as intended (No)	Dyadic sessions	18	4%
	Triadic sessions	18	2%
**Adherence to process elements or competence**			
Repeat/paraphrase/probing questions	Dyadic sessions	18	99%
	Triadic sessions	18	99%
Skipped opportunities	Dyadic sessions	18	0.00
	Triadic sessions	18	0.00
Pacing (rating) 1 = too short, 2 = adequate, 3 = too long	Dyadic sessions	18	2.16 (0.38)
	Triadic sessions	18	2.30 (0.48)
Participant Response (rating) 1 (resistant) to 3 (going along)	Dyadic sessions	18	3.00 (0.00)
	Triadic sessions	18	3.00 (0.00)
Overall quality index (rating) 1 (poor) to 5 (excellent)	Dyadic sessions	18	4.30 (0.36)
	Triadic sessions	18	4.50 (0.23)

## Discussion

The ATTACH™ Fidelity Assessment checklist was developed to evaluate facilitators' adherence and competence in implementing an evidence-based, psycho-educational parenting program to high-risk families. We found that facilitators adhered to the program content and process fidelity close to 100%. They adequately paced or completed the sessions within 30–45 min without dragging or rushing and maintained excellent participant responsiveness. The overall quality index of program delivery ranged from 4 to 5 on a 5-point Likert scale (1 = poor, 5 = excellent). Coders for the ATTACH™ sessions helped to further refine the checklist by requesting minor changes.

Supported by previous research, we developed the ATTACH™ fidelity checklist to measure the elements of adherence to content and process, as well as participant responsiveness (44, 47, 55, 60, 62–64). Overall pacing of program delivery, used in our checklist, has never been included in previous parenting program fidelity checklists, to our knowledge. This item was adapted from fidelity measures of psycho-educational interventions (66). While overall fidelity of program delivery was only rated in a few parenting programs and mostly based on content validity (14, 55, 57), we created an overall quality index rating from psycho-educational intervention fidelity assessment based on both content and process elements (66, 70, 76–78). As ATTACH™ is a psycho-educational parenting program, we deemed it important to include both pacing and overall assessment items to ensure we capture the essence of program delivery.

As we reviewed in Table 1, most of the extant fidelity checklists of the parenting programs have limitations as they are developed to minimize or eliminate the possibility that a practitioner will miss or fail to perform one or more steps or actions. However, assessing adherence to process elements or competence by simple using “Yes” and “No” categories may not be the best answer to meet all needs, specifically in case of psycho-educational interventions such as ATTACH™ (44, 57, 66). We therefore also applied insights from other, non-parenting intervention fidelity measures (such as Song et al. and Miller et al.) to fully comprehend and assess process elements or competence. We also provided an overall quality index of the intervention delivery, as supported by other fidelity literature of parenting interventions and non-parenting interventions (14, 57, 66, 68).

The ATTACH™ fidelity checklist will be specifically important in evaluating the efficacy of the program on outcomes, exploring ways to improve the program, and learning how to overcome challenges and barriers to program implementation (8, 55). The checklist may also improve the efficiency of training facilitators and systematic evaluation of their adherence and competence (79). Assessing competence of facilitators in program delivery is crucial because a facilitator may implement all the content of the program, but in a non-prescribed manner that can result in low efficacy of the program (14). Any deviated delivery of a program element and not using positive communication behaviors can be discouraged and corrected by assessing the program sessions by using our fidelity checklist.

Although only two facilitators delivered the ATTACH™ sessions evaluated here, the approach of monitoring and assessment is applicable to multiple facilitators. While we developed the fidelity checklist for training and monitoring fidelity in research contexts, it has not been evaluated in a clinical setting. Although inter-rater reliability was high (80), only two highly trained coders were assessed. Inter-rater reliability with clinical raters and a larger sample of sessions is needed to confirm these results. A second limitation is the lack of variability in some of the scores (e.g., Participant Responsiveness) of fidelity. Although this indicates overall good adherence and competence in delivering the ATTACH™ intervention, which is a positive finding, lack of variability in fidelity items limit the ability to understand the distinct items that may influence outcomes (12). This should be discussed as a limitation and potential future direction for research. Further work may focus on greater variability in a sample of community-based facilitators, suggesting that it might be valuable to retain these measures until the measure can be validated in a community implementation setting.

Questions remain concerning whether variations in program implementation should be evaluated as part of program fidelity by taking participants' responsiveness into account as moderators of the program effects on outcomes, or both. In future research, weights for different elements may be taken into consideration when critical and core elements have been identified; however, this could be difficult to implement in clinical settings. There are important cost considerations in using fidelity measures (81), as we found that evaluation of an hour-long single session consumed up to 2.5 h for coding and 30 min for scoring by an experienced coder/rater. Other fidelity measures (e.g., 58, 60) did not involve rating a full intervention session, instead they rated a session segment that took as little as 5 min, as a solution to potential time and cost issues in the discussion. Given the creation and testing phase of ATTACH™, we did not know whether significant differences exist between the major program elements (video feedback and RF exercises). Thus, we treated all steps equally in importance and considered them to be delivered within a specific range of time set for the intervention within the protocol.

In future, an extension of this research would examine links between program fidelity and targeted outcomes (e.g., increased fidelity scores may yield more favorable effects or outcomes) to help ascertain if the benefits outweigh the costs (62, 82, 83). This further step would provide evidence for validity of the ATTACH™ fidelity checklist. Nonetheless, measuring fidelity is arguably even more important as interventions are implemented in community settings outside of research studies, with less control and oversight over fidelity, and decreases in fidelity are linked to reduced intervention outcomes [e.g., (84)].

## Conclusion

The program content elements are unique to the ATTACH™ program; therefore, the process of developing and evaluating a fidelity checklist is specifically described for the ATTACH™ parenting program. However, the ATTACH™ fidelity checklist described in this paper, may inform and be adapted to fidelity evaluation tools for other parenting program. High fidelity scores in ATTACH™ sessions provided an evidence of validity for the measure. Establishing treatment differentiation between the ATTACH™ and other manualised interventions would provide another future direction or next step in validation of this measure (33). We have designed a reliable measure and shown how nurses may operationalize program adherence and competence to evaluate facilitators' adherence and competence so that elements of the implementation of parenting programs are more evident. Our evaluation showed the ATTACH™ intervention fidelity was high. Development of checklists like the ATTACH™ fidelity assessment checklist enables the systematic evaluation of program delivery and identification of therapeutic components that enable targeted efforts at improvement.

## Data Availability Statement

The original contributions presented in the study are included in the article/supplementary material, further inquiries can be directed to the corresponding author/s.

## Ethics Statement

The studies involving human participants were reviewed and approved by Conjoint Health Research Ethics Board, University of Calgary, Calgary, Canada. The patients/participants provided their written informed consent to participate in this study.

## Author Contributions

LA: formulated the research question and oversaw all aspects of manuscript preparation from literature review, found all of the relevant articles for fidelity assessment checklist development, developed and refined the checklist with the help of KB and NL, conducted the data analysis, interpreted the results, and undertook writing and submission. KB: helped oversee the research question, literature review, development of checklist, interpretation of the results, and writing. CE: helped oversee the literature review and reviewed the relevant articles, interpreted the results, and writing. MH: helped review the relevant articles, development and refinement of checklist, interpreted the results, and writing. NL: helped formulate the research question, oversaw the literature review and reviewed the relevant articles, helped in the development and refinement of fidelity checklist, interpretation of the results, and writing. All authors have read and approved the final manuscript.

## Conflict of Interest

The authors declare that the research was conducted in the absence of any commercial or financial relationships that could be construed as a potential conflict of interest.
